# Interleukin 6 and 10 Serum Levels and Genetic Polymorphisms in Children with Down Syndrome

**DOI:** 10.1155/2018/6539548

**Published:** 2018-08-16

**Authors:** Marlon Fraga Mattos, Patrícia Matos Biselli-Chicote, Joice Matos Biselli, Thiago Luís da Silva Assembleia, Eny Maria Goloni-Bertollo, Érika Cristina Pavarino

**Affiliations:** ^1^Unidade de Pesquisa em Genética e Biologia Molecular, Faculdade de Medicina de São José do Rio Preto, São José do Rio Preto, SP, Brazil; ^2^Instituto de Biociências Letras e Ciências Exatas, Universidade Estadual Paulista, São José do Rio Preto, SP, Brazil

## Abstract

Immunological impairment is a condition that is often observed in individuals with Down syndrome (DS). The immune response is modulated by pro- and anti-inflammatory cytokines whose expressions could be influenced by genetic polymorphisms. The present study was aimed at evaluating the frequencies of -174G>C, -572G>C, and -597G>A polymorphisms in the interleukin 6 (IL-6) gene and -592C>A, -1082A>G, and -819C>T polymorphisms in the IL-10 gene and the IL-6 and IL-10 serum levels in healthy individuals with and without DS. The authors also aimed to investigate the impact of the genotypes on the interleukin concentrations. The genetic polymorphisms were investigated in 200 DS individuals and 200 controls without DS. The serum measurement of IL-6 and IL-10 was performed in a subgroup (54 cases and 54 controls) by enzyme-linked immunosorbent assay (ELISA). The frequencies of the polymorphisms and haplotypes evaluated were not different between individuals with and without DS. IL-10 concentration was higher in DS children but was not influenced by IL-10 gene polymorphisms. IL-6 genotypes had no influence on IL-6 serum levels. The IL-10 serum levels are increased in DS individuals, but IL-10 polymorphisms are not the main factors that influence the IL-10 expression in DS.

## 1. Introduction

Immunological impairment is a condition often observed in individuals with Down syndrome (DS), which presents an increased susceptibility to bacterial and viral infections and a high frequency of hematologic and autoimmune disorders [1–3]. The immune response is modulated by anti-inflammatory and proinflammatory cytokines, which regulate T cell differentiation. Regulatory cytokines include interleukins (IL), interferons (IFN), tumor necrosis factors (TNF), and growth factors [4].

Interleukin 6 (IL-6) is a proinflammatory cytokine produced by leukocytes, adipocytes, endothelial cells, fibroblasts, and myocytes. IL-6 induces the production of mediators for the release of cytokines such as TNF and IL-1, which drive the inflammatory reaction [5]. The immune system uses anti-inflammatory mechanisms to prevent the exacerbation of inflammatory processes caused by proinflammatory molecules and avoid tissue damage and restore the homeostasis [6]. IL-10 is an important immunoregulatory and anti-inflammatory cytokine secreted by macrophages, monocytes, dendritic cells, T helper 1 (Th1) and Th2 lymphocytes, B lymphocytes, cytotoxic T cells, and mast cells [7]. IL-10 stimulates the activation, proliferation, and differentiation of B cells [6] and participates in the control of the inflammatory response [8]. An imbalance between pro- and anti-inflammatory cytokines avoids the adequate function of the immune system. In DS, alteration of cytokine levels has been observed [9–15], and it can lead to immune deficiency.

The single-nucleotide polymorphisms within the promoter region -597G>A, -572G>C, and -174G>C of the IL-6 gene and -1082G>A, -829C>T, and -592C>A of the IL-10 gene were described [16–18] and can be involved in the modulation of inflammatory responses and susceptibility to inflammatory disorders [17, 19–23].

Considering the immunological impairment in DS individuals, we aimed to determine the prevalence of the polymorphisms -597G>A (rs1800797), -572G>C (rs1800796), and -174G>C (rs1800795) in the IL-6 gene and -1082A>G (rs1800896), -829C>T (rs1800871), and -592C>A (rs1800872) in the IL-10 gene and the serum level of IL-6 and IL-10 in these individuals in comparison with individuals without DS. We also aimed to evaluate the association between these polymorphisms and IL-6 and IL-10 concentrations.

## 2. Methods

The study was approved by the Research Ethics Committee of Faculdade de Medicina de São José do Rio Preto (FAMERP) (number 427.782).

### 2.1. Subjects

The study included 200 children with DS (mean age = 4.3 years, 108 males and 95 females) from the General Genetics Outpatient Service of Hospital de Base, São José do Rio Preto, SP, Brazil, and 200 individuals without DS (mean age = 4.3 years, 103 males and 97 females) from the Pediatric Service of the Hospital de Base de São José do Rio Preto, SP, Brazil; all the parents of individuals who participated in the study signed an informed consent. Only individuals who were without leukemia or acute or chronic infection and did not receive medication or immunization within 6 weeks from the serum collection were selected for the interleukin dosage. C-reactive protein (CRP) was quantified by electrochemiluminescence, and only samples with a concentration of ≤0.5 mg/dl were included in the analysis.

### 2.2. Polymorphism Analysis

DNA was extracted from peripheral blood [24]. Genotyping of IL-10 -1082A>G and -592C>A polymorphisms was performed by polymerase chain reaction (PCR) restriction fragment length polymorphism (RFLP) analysis. Primer sequences used were as follows: forward 5′-TCTGAAGAAGTCCTGATGTC-3′ and reverse 5′-CTCTTACCTATCCCTACTTCC-3′ for the detection of IL-10 -1082A>G polymorphism and forward 5′-GGTGAGCACTACCTGACTAGC-3′ and reverse 5′-CCTAGGTCACAGTGACGTGG-3′ for the detection of IL-10 -592C>A polymorphism. PCR products were digested by the restriction enzymes MnlI (New England Biolabs, Ipswich, MA) and RsaI (New England Biolabs, Ipswich, MA) for -1082A>G and -592C>A, respectively. The digested products were analyzed on 2.5% agarose gel.

Genotyping of IL-10 -819C>T, IL-6 -174G>C, IL-6 -572G>C, and IL-6 -597G>A was performed using TaqMan SNP Genotyping Assays (C_1747362_10, C_1839697_20, C_11326893_10, and C_1839695_20, Applied Biosystems, Foster City, CA), following the manufacturer's instructions.

### 2.3. Quantification of IL-10 and IL-6 Serum Levels

The quantification of IL-10 and IL-6 serum levels was performed in a subgroup composed of 54 individuals with DS (three wild-type homozygous, three heterozygous, and three mutated homozygous for each polymorphism) and 54 individuals without DS (three wild-type homozygous, three heterozygous, and three mutated homozygous for each polymorphism). The interleukin concentrations were also evaluated according to the genotype combination and haplotypes. IL-6 and IL-10 quantification was performed using the Novex ELISA kit (Life Technologies, Carlsbad, CA), following the manufacturer's instructions, and they were analyzed on the Multiskan FC Microplate Photometer (Thermo Scientific, Waltham, MA) at 450 nm.

### 2.4. Statistical Analysis

Allele frequencies were evaluated for the Hardy-Weinberg (HWE) equilibrium by the chi-square test using the BioEstat software version 5.0. SNPStats program (http://bioinfo.iconcologia.net/SNPstats_web) for the analysis of the genotype distribution between the groups by logistic regression in the codominant, dominant, and recessive models.

The results were presented as odds ratio (OR) at 95% confidence interval (95% CI). Haplotype analysis was performed using Haploview software, version 4.2. A comparison of IL-6 and IL-10 serum levels between the groups was performed by the Mann–Whitney test. Analysis of interleukin concentrations in relation to the genotypes was performed using the Kruskal-Wallis or Mann–Whitney test with GraphPad Prism software version 6.0. The error accepted was 5%.

## 3. Results

### 3.1. Polymorphisms in DS and Control Groups

The genotype distribution of IL-6 -174G>C (rs1800795), IL-6 -572G>C (rs1800796), and IL-6 -597G>A (rs1800797) polymorphisms was in accordance with the Hardy-Weinberg equilibrium (HWE) in the DS (*P* = 0.68 for -174G>C, *P* = 0.48 for -572G>C, and *P* = 0.68 for -597G>A) and control (*P* = 0.71 for -174G>C, *P* = 0.51 for -572C>G, and *P* = 1 for -597G>A) groups.

The genotype frequencies of IL-10 -592C>A polymorphism did not differ from those that we would expect under HWE in the DS (*P* = 0.75) and control (*P* = 0.19) groups. IL-10 -819C>T polymorphism was in accordance with HWE only in the DS group (*P* = 0.76). In the control group, the genotype frequencies deviated from HWE expectations (*P* = 0.036). The genotype frequencies of IL-10 -1082A>G presented HWE deviation in DS (*P* = 0.026) and control (*P* < 0.0001) groups.

There was no significant difference in genotype distribution between the groups (Table 1). Haplotype analyses were conducted to evaluate the combined effect of the polymorphisms. The IL-6 polymorphisms were in strong linkage disequilibrium, as were the IL-10 polymorphisms. Frequencies of alleles and haplotypes of IL-6 and IL-10 polymorphisms were not statistically different between the groups (*P* > 0.05).

### 3.2. IL-6 and IL-10 Serum Levels in DS and Control Groups

**Figure 1:**
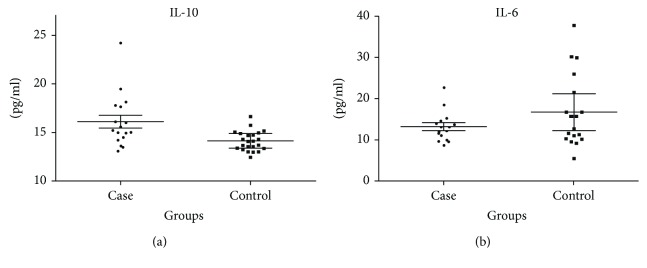
Interleukin concentrations between the groups. (a) IL-10 serum levels in DS (16.10 pg/ml) and control (14.09 pg/ml) groups. Mann–Whitney test (*P* = 0.002). (b) IL-6 serum levels in DS (13.03 pg/ml) and control (15.69 pg/ml) groups. Mann–Whitney test (*P* = 0.69). The bars represent median with interquartile variation (25th percentile and 75th percentile).

IL-10 serum levels were significantly increased in DS children compared to those without DS (*P* = 0.0019; Figure 1(a)). IL-6 serum levels did not differ between the DS and control groups (*P* > 0.05; Figure 1(b)).

IL-06 and IL-10 concentrations were evaluated in relation to the genetic polymorphisms; however, IL-6 and IL-10 polymorphisms showed no effect on these interleukin serum levels (Table 2). The polymorphic homozygous genotype for IL-6 -572G>C polymorphism was not detected in this subgroup, and so, the statistical analyses according to the genetic models of the IL-6 were not performed. In this case, the statistic was performed between the wild-type genotype and heterozygous genotype (DS: GG = 13.77 pg/ml versus GC = 10.65 pg/ml (*P* = 0.07); control: GG = 13.67 pg/ml versus GC = 16.73 pg/ml (*P* = 0.99)).

## 4. Discussion

Our findings showed an increase in serum levels of the cytokine IL-10 in children with DS. IL-10 is an anti-inflammatory cytokine [25] which participates in the negative feedback control of inflammatory responses [8]. This cytokine plays a crucial role in the prevention of inflammatory and autoimmune pathologies [6]. IL-10 suppresses the gene expression of other cytokines and chemokines by inhibiting the transcription or reducing the levels of mRNA [8]. Increased IL-10 signaling can prevent the maturation of macrophage and dendritic cells and inhibit the production of proinflammatory cytokine [6]. Thus, excessive IL-10 production can inhibit proinflammatory response to several pathogens, resulting in uncontrolled infection and deficient immune response [6], which are characteristics that are often observed in DS.

The first phase of an innate immune response comprises the classical immune activation [26, 27] characterized by the recruitment of Th1 cytokines such as interferon-*γ* and other proinflammatory cytokines [28, 29]. However, the production of proinflammatory factors can be arrested, and macrophages can produce factors that participate in tissue repair and wound healing, such as anti-inflammatory cytokines [29]. IL-10 and TGF-*β* are associated with the inhibition of proinflammatory activity [29, 30]. This alternative activation during an immune response provides an anti-inflammatory equilibrium to a proinflammatory acute response. The activated macrophages are immunosuppressive and participate in tissue repair and remodeling of the extracellular matrix [28, 29]. However, repair processes can enhance the fibrosis and contribute to the maintenance of disease [29, 31, 32]. An interrelation between inflammatory and regenerative processes was suggested on neurodegeneration related to the pathogenesis of Alzheimer's disease (AD) [33].

Overexpression of IL-10 was previously reported in DS [34–36]. The basal levels of the IL-10 gene expression were observed to be upregulated in DS children [37]. The present study evaluated the expression profile of immune-related genes in DS individuals without current infection and concluded that several genes with relevant functions in immune cells are dysregulated in DS [37]. This could explain the increased susceptibility to bacterial and viral infections and inflammatory disorders in these individuals [38, 39].

In addition to the increased basal levels of IL-10 in DS, studies that evaluated the immune response of DS individuals showed increased levels of IL-10 in the presence of pathogens or inflammatory processes [34, 40]. The immune response to antigens is mediated by proinflammatory cytokines that perform the defense of pathogen invasion [6]. However, an excess of inflammation can disrupt the host's metabolic system. Anti-inflammatory system activation is a mechanism used to avoid tissue damage and restore homeostasis [6]. The inflammatory response to a microbial challenge can be enhanced by down- or overexpression of IL-10. The impairment of IL-10 expression or signaling can result in enhanced removal of pathogens during an acute infection but also can contribute to an exacerbated inflammatory response, resulting in immunopathology and tissue damage [6].

Differential expression of inflammation-related genes was observed in healthy children with DS [41]; IL-10 levels were higher in DS individuals with AD and also in those with DS without a clinically relevant cognitive decline [36]. In a previous study, IL-10 levels from the DS group did not differ from those from the control group; however, it was significantly lower in DS patients than in patients with intellectual disability [42]. It is believed that soluble amyloid precursor protein and several forms of *β*-amyloid peptides lead to the activation of the signaling for an innate immune response in the brain [43]. Studies have shown that a proinflammatory state can reduce *β*-amyloid accumulation in mouse models [44–50], and the high concentrations of IL-10 contribute to reduced amyloid-*β* phagocytosis by microglia and amyloid-*β* deposition [51], which is observed in DS individuals.

Interleukin 6 (IL-6) plasma levels were also higher in subjects with DS and AD-related symptoms [36]. A negative correlation was found between IL-6 levels and cognitive decline [36]. Studies have found increased levels of IL-6 in DS [36, 52], although others have observed opposite results [34] or no significant alterations [40]. We did not observe differential concentrations of IL-6 between individuals with DS and those without the syndrome in this study.

IL-6 stimulates T and B cell immune responses upon encountering antigen components, triggering an acute inflammatory response and haematopoiesis [5, 53]. It is important to emphasize that we evaluated individuals with no infection at the moment of sample collection; therefore, we evaluated the basal levels of IL-6. Perhaps, IL-6 is more significantly related to the immune response in these individuals, and its abnormal production occurs after the contact with an antigen. IL-6 concentrations were significantly higher in children with DS upon stimulation with influenza A virus, which reinforces its role in the immune response [54]. Overexpression of proinflammatory cytokines, such as IL-6, could result in injury to healthy tissue [55] and overinflammation, leading to neuronal dysfunction and consequent deterioration of the neurons, as observed in AD progression [56].

The levels of cytokines can be determined by genetic polymorphisms in the promoter region of interleukin genes [7, 17, 57–60]. In this study, we did not find an association between all IL-6 or IL-10 polymorphisms evaluated and the concentrations of these interleukins in both groups of DS individuals and those without the disease.

The polymorphisms -1082 A>G, -592C>A, and 819C>T in the IL-10 gene seem to modulate the IL-10 levels in different disorders, such as cancer [21], tuberculosis [19], systemic lupus erythematosus [20], and rheumatoid arthritis [22], but the results are controversial. To the best of our knowledge, there is no study relating these genetic alterations to IL-10 concentrations in DS until now.

IL-6 -174G>C, -572G>C, and -597G>A polymorphisms have been investigated on the levels of this cytokine in inflammatory diseases such as systemic-onset juvenile chronic arthritis [17], age-related macular degeneration [61], type 2 diabetes [62], age-related macular degeneration [63], systemic lupus erythematosus [20], cognitive impairment [63], and dementia [64]. The results are different according to the disease.

The frequencies of the IL-10 and IL-6 polymorphisms evaluated here did not differ between DS individuals and those without the syndrome. To our knowledge, this is the first study to investigate these genetic alterations in DS. Polymorphisms in the IL-10 gene have been associated with some immune-related diseases [54] such as asthma [65, 66], systemic lupus erythematosus [67–69], Crohn's disease [70, 71], rheumatoid arthritis [22, 72–74], tuberculosis [19, 75], type 2 diabetes [76], and several types of cancer [77–80]. Alterations in the IL-6 gene were also related to inflammatory changes [17, 18, 23, 81–84], including AD [83, 84]. However, IL-6 -572G>C was associated with protection in osteoarthritis [85] and erythematosus systemic lupus [86]. As previously mentioned, there are no data in the literature on the role of these polymorphisms in DS.

## 5. Conclusion

In conclusion, the IL-10 -1082A>G, IL-10 -592C>A, IL-10 -819C>T, IL-06 -597G>A, IL-06 -174G>C, and IL-06 -572G>C polymorphisms have no effect on IL-10 and IL-6 concentrations in DS individuals and individuals without the syndrome evaluated in this study. The levels of IL-10 are increased in DS individuals, but the polymorphisms in the IL-10 gene are not the main factors that drive the overexpression of IL-10 in DS.

## Figures and Tables

**Table 1:** Genotype distribution of IL-6 and IL-10 polymorphisms in DS and control groups.

Models	Genotype	Control (*N* (%))	DS (*N* (%))	OR (95% CI)	*P* value	Genotype	Control (*N* (%))	DS (*N* (%))	OR (95% CI)	*P* value
	IL-6 -597G>A					IL-10 -1082A>G				
Codominant	GG	107 (53.5)	122 (61)	1.00	0.22	AA	104 (52)	95 (47.5)	1.00	0.44
	GA	79 (39.5)	70 (35)	1.28 (0.84–1.93)		AG	63 (31.5)	75 (37.5)	0.76 (0.49–1.18)	
	AA	14 (7)	08 (4)	1.99 (0.80–4.92)		GG	33 (16.5)	30 (15)	1.01 (0.57–1.78)	
Dominant	GG	107 (53.5)	122 (61)	1.00	0.14	AA	104 (52)	95 (47.5)	1.00	0.36
	GA/AA	93 (46.5)	78 (39)	1.35 (0.91–2.01)		AG/GG	96 (48)	105 (52.5)	0.83 (0.56–1.23)	
Recessive	GG/GA	186 (93)	192 (96)	1.00	0.19	AA/AG	167 (83.5)	170 (85)	1.00	0.67
	AA	14 (7)	08 (4)	1.80 (0.74–4.40)		GG	33 (16.5)	30 (15)	1.12 (0.66–1.93)	

	IL-6 -174G>C					IL-10 -592C>A				
Codominant	GG	108 (54)	120 (60)	1.00	0.41	CC	98 (49)	91 (45.5)	1.00	0.70
	GC	80 (40)	72 (36)	1.22 (0.81–1.85)		CA	78 (39)	86 (43)	0.84 (0.55–1.27)	
	CC	12 (6)	08 (4)	1.66 (0.65–4.21)		AA	24 (12)	23 (11.5)	0.96 (0.51–1.83)	
Dominant	GG	108 (54)	120 (60)	1.00	0.24	CC	98 (49)	91 (45.5)	1.00	0.47
	GC/CC	92 (46)	80 (40)	1.27 (0.85–1.89)		CA/AA	102 (51)	109 (54.5)	0.86 (0.58–1.28)	
Recessive	GG/GC	188 (94)	192 (96)	1.00	0.36	CC/CA	176 (88)	177 (88.5)	1.00	0.88
	CC	12 (6)	08 (4)	1.53 (0.61–3.82)		AA	24 (12)	23 (11.5)	1.05 (0.57–1.93)	

	IL-06 -572G>C					IL-10 -819C>T				
Codominant	GG	155 (77.5)	156 (78)	1.00	0.37	CC	84 (42)	83 (41.5)	1.00	0.13
	GC	41 (20.5)	43 (21.5)	0.97 (0.6–1.57)		CT	80 (40)	94 (47)	0.84 (0.55–1.28)	
	CC	04 (2)	01 (0.5)	4.07 (0.45–36.78)		TT	36 (18)	23 (11.5)	1.54 (0.84–2.82)	
Dominant	GG	155 (77.5)	156 (78)	1.00	0.88	CC	84 (42)	83 (41.5)	1.00	0.91
	GC/CC	45 (22.5)	44 (22)	1.04 (0.65–1.66)		CT/TT	116 (58)	117 (58.5)	0.98 (0.66–1.45)	
Recessive	GG/GC	196 (98)	199 (99.5)	1.00	0.16	CC/CT	164 (82)	177 (88.5)	1.00	0.07
	CC	04 (2)	01 (0.5)	4.10 (0.45–36.96)		TT	36 (18)	23 (11.5)	1.69 (0.96–2.97)	

DS: Down syndrome; OR: odds ratio; CI: confidence interval.

**Table 2:** IL-6 and IL-10 serum levels in relation to the genotypes.

Models	Genotype	DS (pg/ml)	*P* value	Control (pg/ml)	*P* value	Genotype	DS (pg/ml)	*P* value	Control (pg/ml)	*P* value
	IL-10 -1082A>G					IL-6 -597G>A				
Codominant	AA	15.07	0.82	13.60	0.69	GG	11.52	0.49	16.22	0.12
	AG	15.02		14.33		GA	13.59		19.12	
	GG	16.84		13.71		AA	12.17		11.00	
Dominant	AA	15.07	0.91	13.60	0.59	GG	11.52	0.27	16.22	0.89
	AG/GG	16.08		14.30		GA/AA	13.32		12.80	
Recessive	AA/AG	15.02	0.62	14.09	0.75	GG/GA	13.04	0.90	16.74	0.07
	GG	16.84		13.71		AA	12.17		11.00	

	IL-10 -592C>A					IL-6 -174G>C				
Codominant	CC	16.84	0.57	14.03	0.94	GG	11.52	0.49	16.22	0.12
	CA	15.18		14.08		GC	13.59		19.12	
	AA	14.72		14.27		CC	12.17		11.00	
Dominant	CC	16.84	0.47	14.03	0.94	GG	11.52	0.27	16.22	0.89
	CA/AA	15.02		14.08		GC/CC	13.32		12.80	
Recessive	CC-CA	15.58	0.31	14.08	0.90	GG/GC	13.04	0.90	16.74	0.07
	AA	14.72		14.27		CC	12.17		11.00	

	IL-10 -819C>T					IL-6 -572G>C				
Codominant	CC	16.84	0.57	14.03	0.94	GG	13.77	0.07	13.67	0.99
	CT	15.18		14.08		GC	10.65		16.73	
	TT	14.72		14.27		—^∗^	—		—	
Dominant	CC	16.84	0.47	14.03	0.90	GG	—	—	—	—
	CT/TT	15.02		14.08		GC/−^∗^				
Recessive	CC/CT	15.58	0.31	14.08	0.90	GG/GC	—	—	—	—
	TT	14.72		14.27		—^∗^				

^∗^Genotype was not found. DS: Down syndrome.

## Data Availability

The statistical data used to support the findings of this study are included within the article.
